# Supplementary material for: Selective catalytic oxidation of ammonia to nitric oxide via chemical looping
Source: Nat Commun. 2022 Feb 7;13:718. doi: 10.1038/s41467-022-28370-0 (PMC8821626; doi:10.1038/s41467-022-28370-0)
Supplement: Supplementary file 1 — Supplementary Information [file 41467_2022_28370_MOESM1_ESM.pdf]

Supplementary Information

Selective catalytic oxidation of ammonia to nitric oxide via chemical looping

Chongyan Ruan[1,2,‡], Xijun Wang[2,‡], Chaojie Wang[1,3], Lirong Zheng[4], Lin Li[1], Jian Lin[1], Xiaoyan Liu[1], Fanxing Li*[2] and Xiaodong Wang*[1]

[1] CAS Key Laboratory of Science and Technology on Applied Catalysis, Dalian Institute of Chemical Physics, Chinese Academy of Sciences, Dalian 116023, China.

[2] Department of Chemical and Biomolecular Engineering, North Carolina State University, Raleigh, NC, USA.

[3] University of Chinese Academy of Sciences, Beijing, 100049, China.

[4] Institute of the High Energy Physics, Chinese Academy of Sciences, Beijing 100049, China.

[‡] These authors contributed equally: Chongyan Ruan, Xijun Wang

***Corresponding Authors**: xdwang@dicp.ac.cn; fli5@ncsu.edu

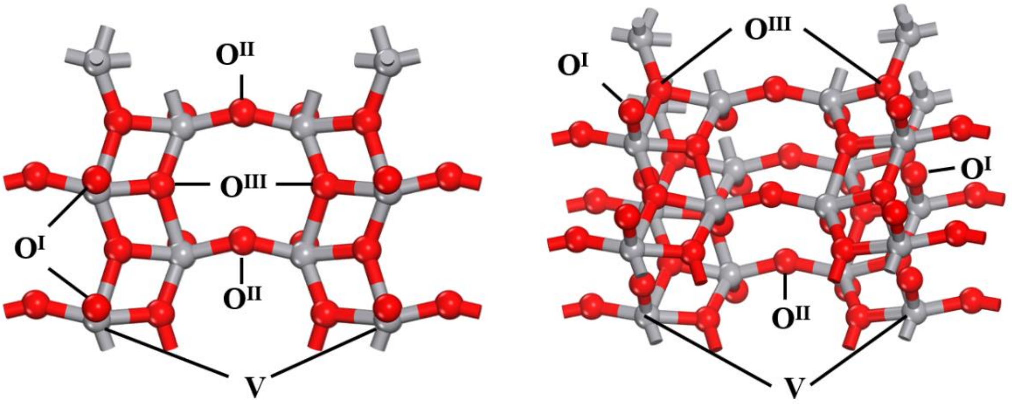

Fig. S1 Top (left) and side (right) view of the unit cell of V_2O_5(001) surface.

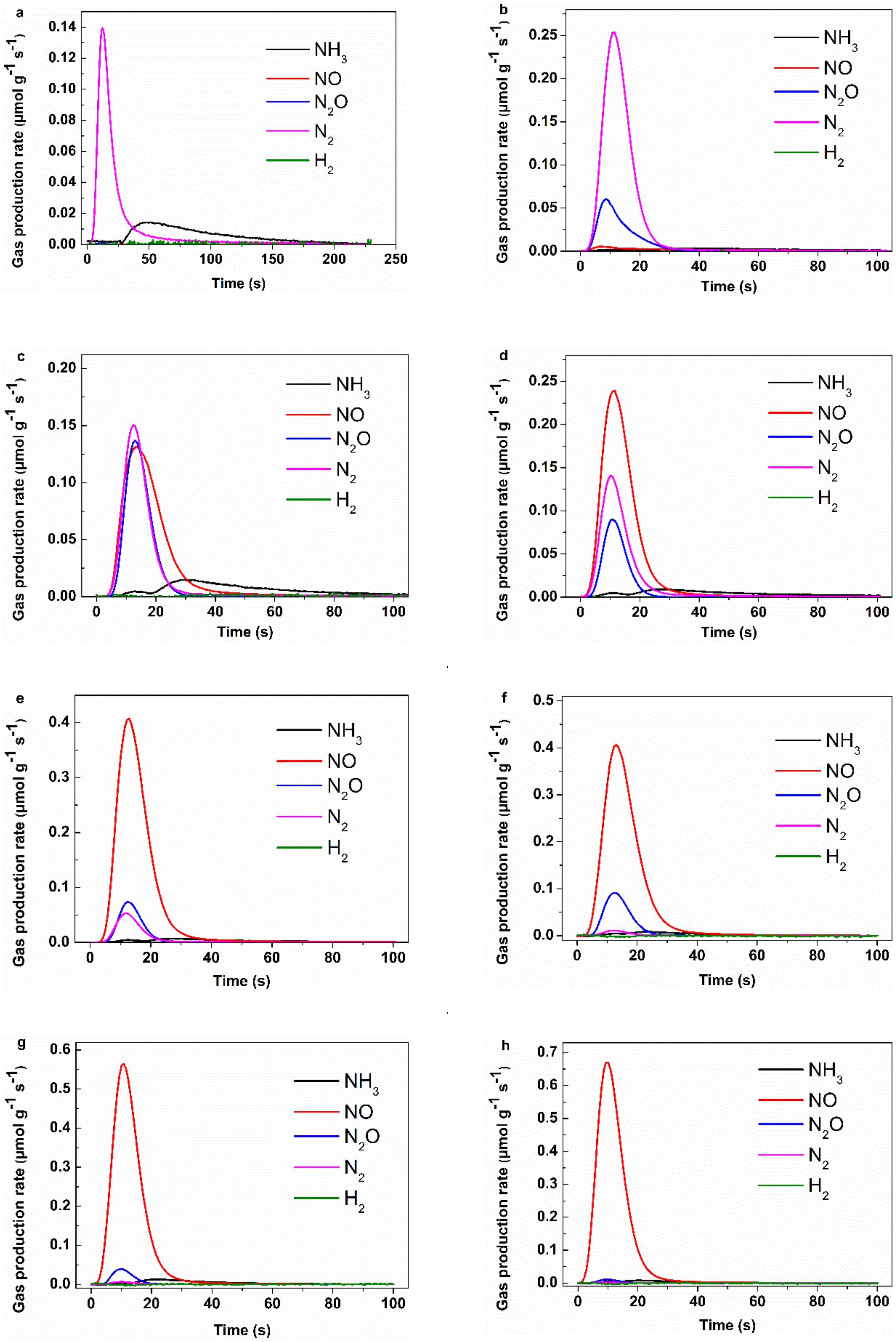

Fig. S2 Normalized transient products (NO, N_2O, N_2, H_2) evolution rates upon NH_3 injection over V_2O_5 at (a) 300 ºC, (b) 400 ºC, (c) 500 ºC, (d) 550 ºC, (e) 580 ºC, (f) 600 ºC, (g) 630 ºC, and (h) 650 ºC in chemical looping NH_3 oxidation process.

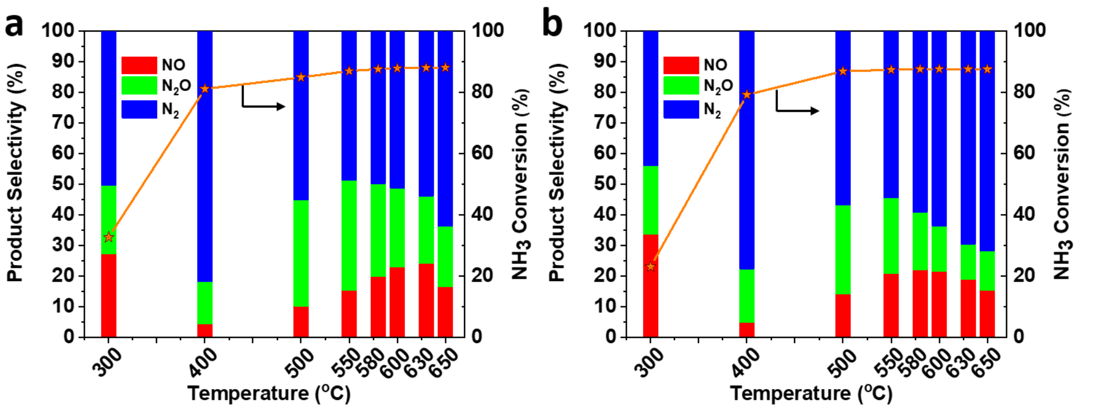

Fig.S3 NH_3 conversion and the distribution of reaction products vs. temperature for steady state NH_3 oxidation process (co-feed mode) with the effective O_2 percentage of (a) 18% (NH_3 1.5 ml/min, O_2 6.59ml/min, Ar 28.5 ml/min), (b) 42% (NH_3 1.5 ml/min, O_2 21.72 ml/min, Ar 28.5 ml/min) at 650 °C.

To investigate the O_2 partial pressure and its influence on production formation for NH_3 oxidation via co-feed mode, the reaction was studied further with the effective O_2 percentage of 18% and 42% with excess O_2 over NH_3. As illustrated in Fig. S3 a, b, the NH_3 conversion and distribution of the reaction products (NO, NO NO) versus temperature are similar. Ammonia conversion increases with increasing temperature and reaches a plateau from 500 to 650 °C. In regard to product selectivity, NO peaks at 300 °C decreases significantly when rising the temperature to 400 °C. Since in the ternary O_2–NH_3–NO system, a competition between the NO reduction by NH_3 and the NH_3 oxidation with O_2 to NO can be expected. The decline in NO formation at 400 °C may due to the secondary reactions involving NO and NH_3. A further increase in temperature led to an increase in NO formation before passing through a maximum. The ammonia conversion and NO selectivity compared less favorable with CLAO process despite using feeds containing an excess of O_2.

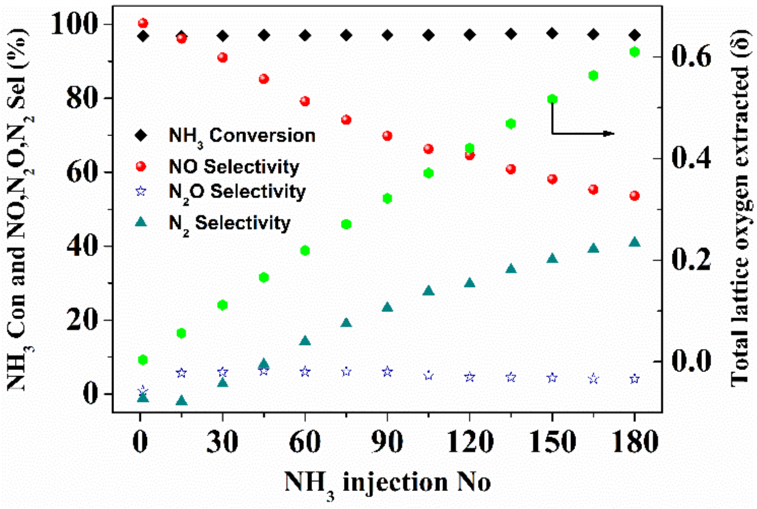

Fig. S4 NH_3 conversion, NO, N_2O, N_2 selectivity as well as total lattice oxygen consumption with 180 NH_3 pulses over V_2O_5 at 650 ºC.

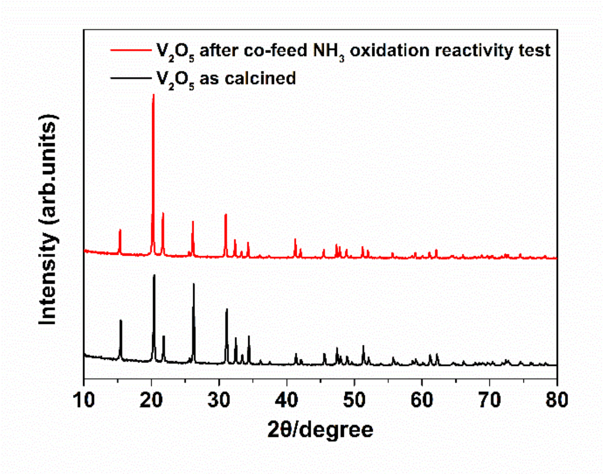

Fig. S5 X-ray powder diffraction patterns of as calcined V_2O_5 and V_2O_5 redox catalyst after activity measurement in conventional co-feed mode.

Fig. S5 shows the XRD patterns of the as prepared V_2O_5 sample before and after NH_3 oxidation reactivity test in the co-feed mode. No apparent structural change was observed for V_2O_5 redox catalyst after the reactivity test.

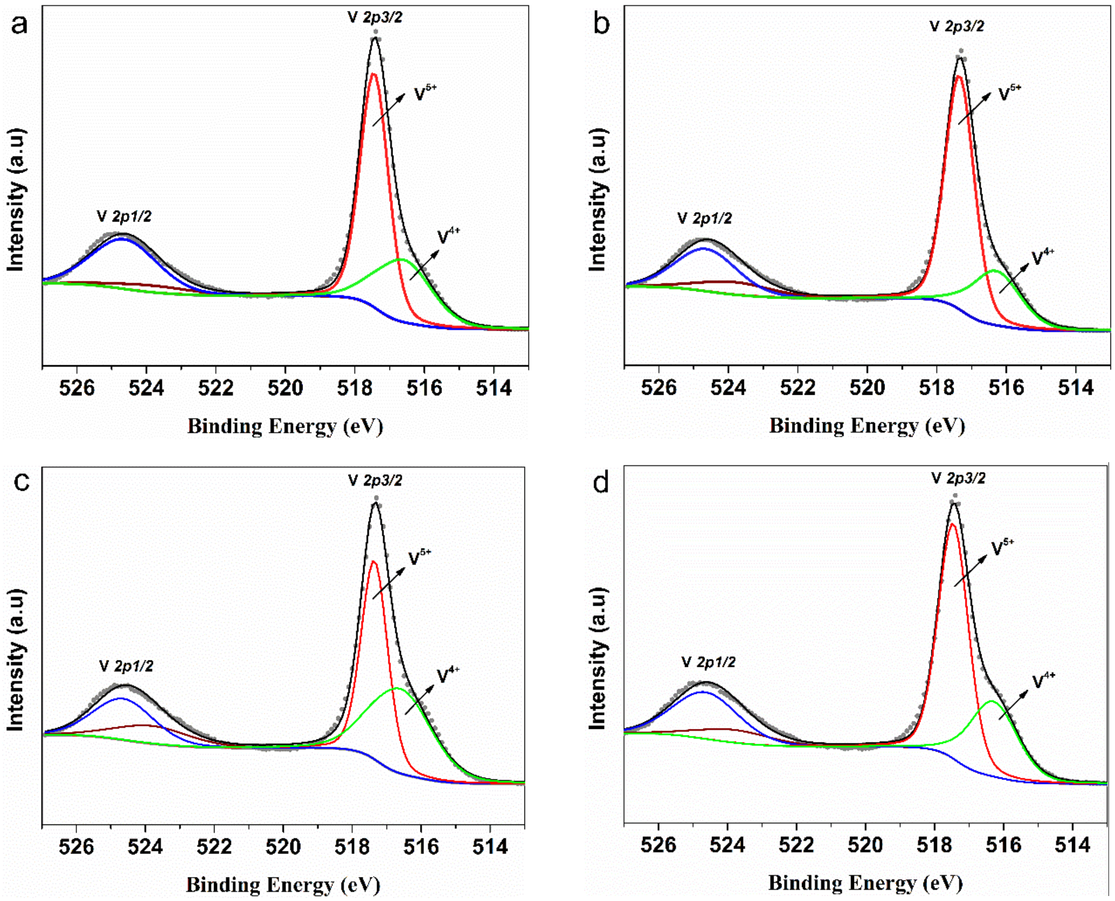

Fig. S6 (a) V 2p XPS spectra of V_2O_5 subjected to (a) 60 pulses, (b) 90 pulses, (c) 120 pulses, and (d) 150 pulses of NH_3 reduction. a.u., arbitrary unit.

Fig. S6a-d shows the V 2p XPS spectra for V_2O_5 subjected to different pulses of NH_3 reduction, i.e., 60, 90, 120 and 150. The deconvolution of the V 2p3/2 and V 2p1/2 peaks exhibits two components corresponding to the V^{4+} and V^{5+} oxidation states, respectively.

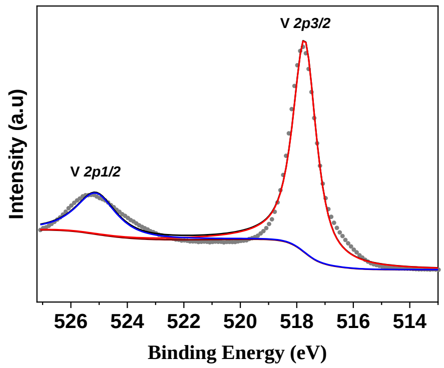

Fig. S7 V 2p XPS spectrum of V_2O_5 redox catalyst after activity measurement in conventional co-feed mode at 650 °C. a.u., arbitrary unit.

The V_2O_5 catalysts after the continuous mode was purged in an inert environment (Ar) followed by cooling to room temperature. Then the reactor was transferred into an argon-filled glove box without exposure to air with both ends sealed. The sample loadings were handled in a glove box filled with argon, and the sample holder was then sealed to minimize the Air exposure. Fig. S7 shows the XPS result for V_2O_5 after NH_3 oxidation reactivity test in the co-feed mode. In agreement with the XRD results, there was no apparent reduction after NH_3 oxidation reaction. A strong peak centered at 517.4 eV along with a broad satellite peak at a higher binding energy was identified, which agrees well with the signature of V^{5+} in V_2O_5.

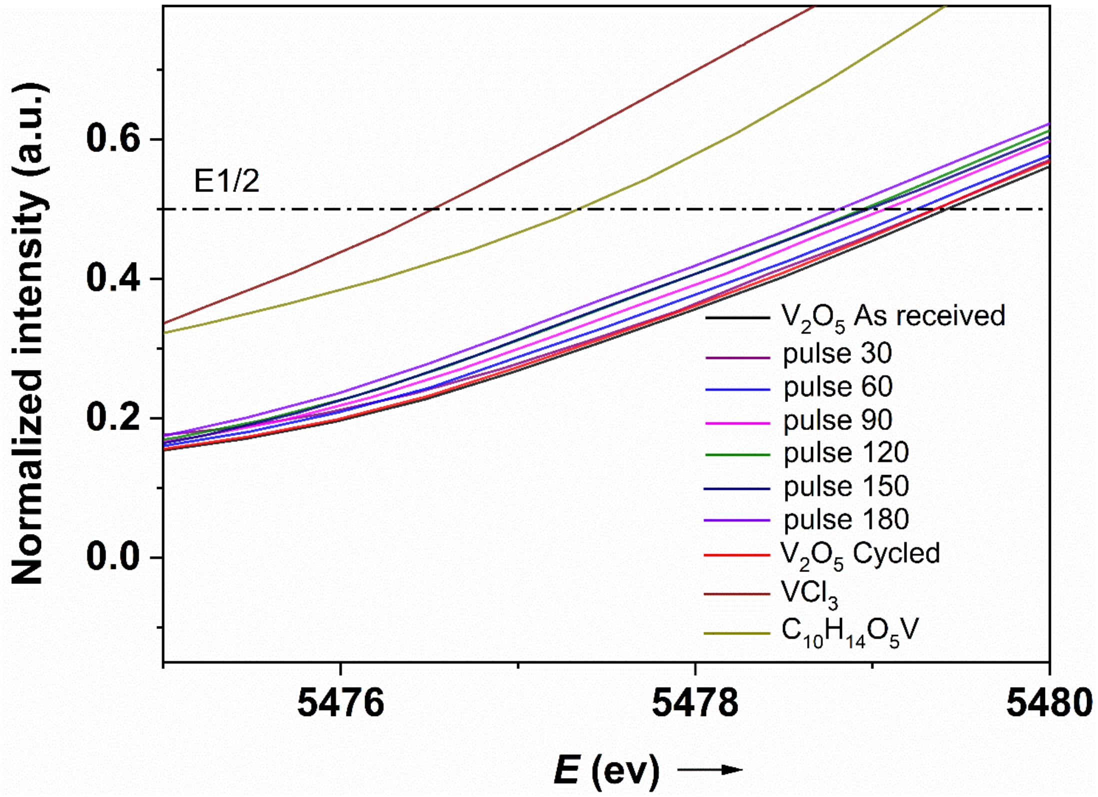

Fig. S8 Absorption edge region of XANES spectra in Fig. 3c highlighting evolution of the absorption edge with increasing NH_3 pulse numbers. The dashed horizontal lines indicates the edge position of E1/2 where the normalized absorption equals 0.5. a.u., arbitrary unit.

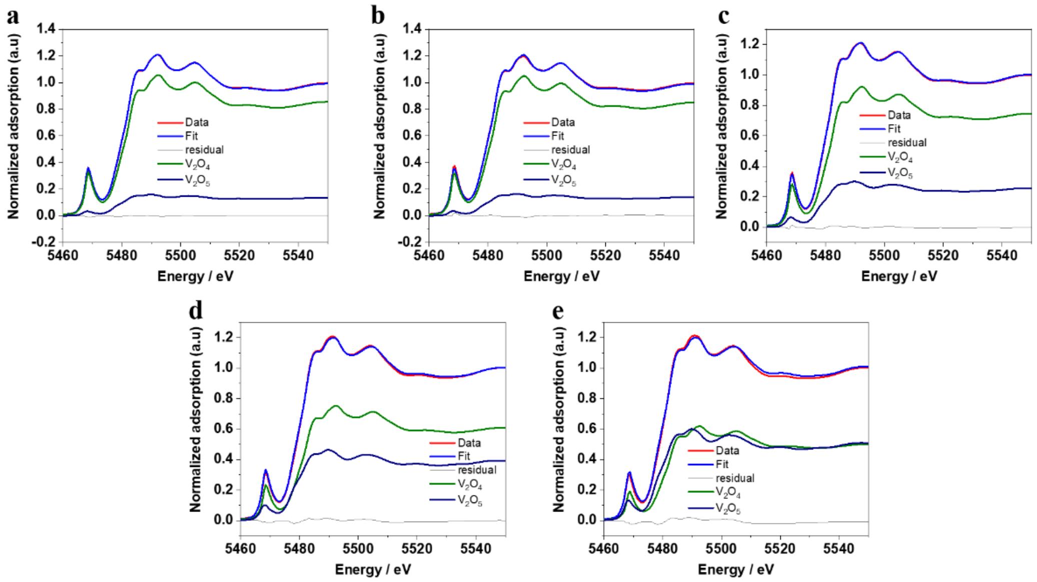

Fig. S9. Normalized V-K edge XANES spectra of V_2O_5 as a function of NH_3 pulses numbers, (a) 60 pulses of NH_3 reduction, (b) 90 pulses of NH_3 reduction, (c) 120 pulses of NH_3 reduction, (d) 150 pulses of NH_3 reduction, (e) 180 pulses of NH_3 reduction, and the resultant linear combination fit with the LCF corrected reference spectra and the corresponding residuals. a.u., arbitrary unit.

Fig. S9 illustrates the linear combination fit (LCF) results using the Athena software. For each spectrum the combination of standards with the lowest residual parameter was chosen. Because the vanadium transitions directly between two states (V^{5+} and V^{4+}), the ratio of V^{5+} to V^{4+} present in each sample could be determined using V_2O_5 and V_2O_4 as the end members in a linear combination fit.

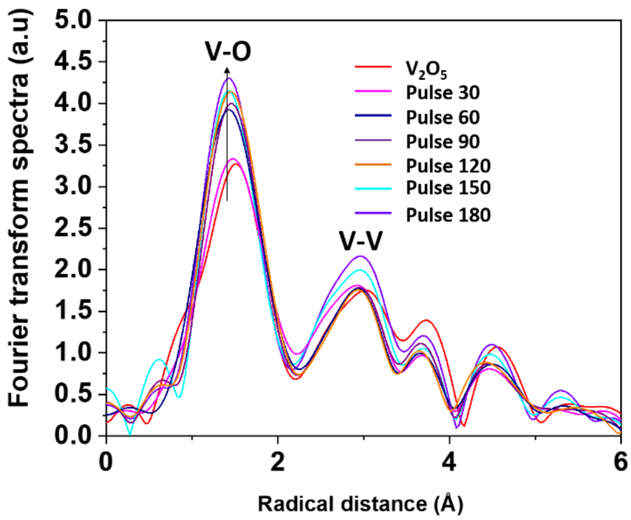

Fig. S10. Fourier transform (FT) of k-weighted V EXAFS spectra of V_2O_5 catalysts as a function of NH_3 pulse numbers. a.u., arbitrary unit.

Fig. S10 shows the Fourier transforms of V *K*-edge EXAFS for V_2O_5 catalysts as a function of NH_3 pulse numbers. The first two peaks at 1.50 Å and 2.85 Å are dominated by single scattered contributions from the first and second coordination spheres of V-O and V-V correlations, respectively. The peaks above 3 Å are complicated by the contributions from a large number of single as well as multiple-scattered contributions from other paths. Hence, only the first and two peaks are discussed here. There is a consistent increase of the amplitude of the V-O coordination sphere with increasing NH_3 pulse numbers, indicating the gradual increase of average coordination number of V-O units with enhanced reduction extent.

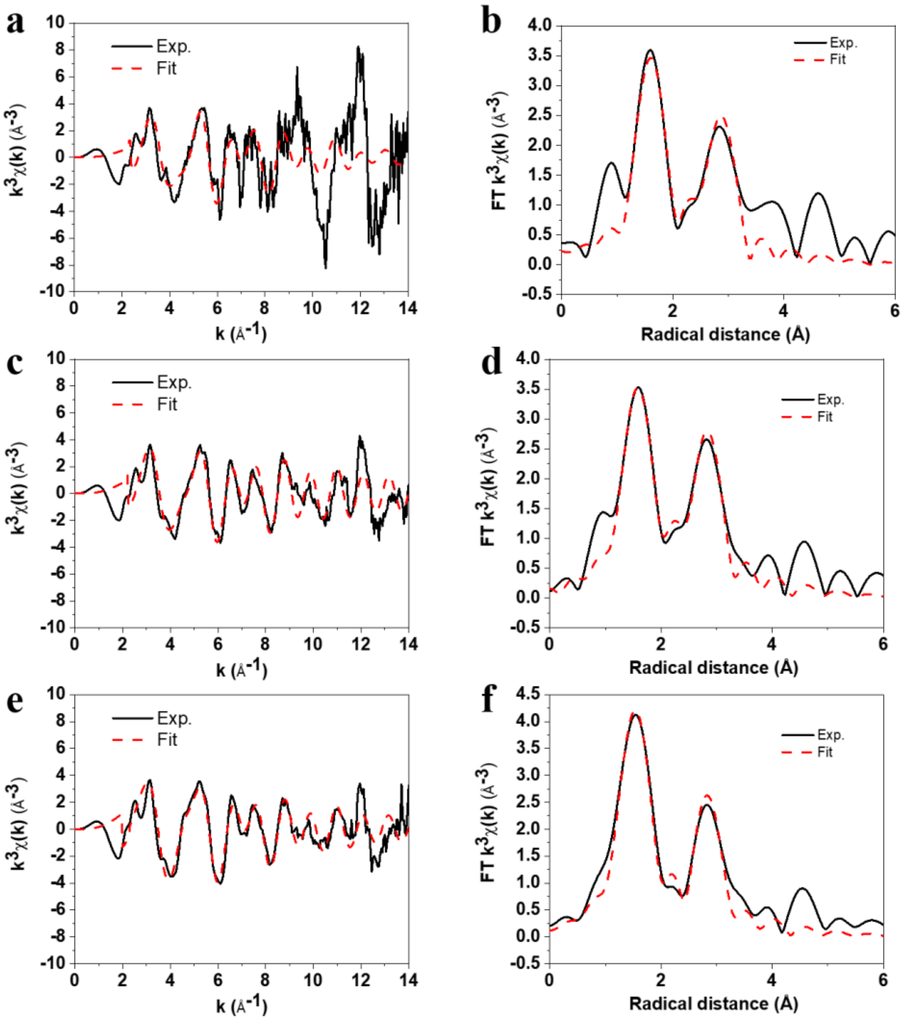

Fig. S11. $k^3\chi(k)$ spectra and Fourier transforms of $k^3\chi(k)$ spectra at the V K-edge of V_2O_5 (a and b), V_2O_5 subjected to 90 pulses of NH_3 reduction (c and d) and V_2O_5 subjected to 180 pulses of NH_3 reduction (e and f) from experiment (—) and fit results (···).

In order to gain quantitative information, fitting of the EXAFS data were performed for initial V_2O_5 and V_2O_5 after 90 and 180 pulses of NH_3 reduction. The results of fitting are presented in Fig. S11, with the best fit parameters summarized in Table S4. As compare to the initial sample, the averaged V-O distance for V_2O_5 catalysts after 90 and 180 pulses of NH_3 reduction increased from 1.80 Å to 1.92 Å. On the contrary, a decrease in the distance of the second coordination sphere of V-V is observed for V_2O_5 catalysts after 90 and 180 pulses of NH_3 reduction. The results are in quality agreement with the XRD observation where V_2O_5 (coordination number, 5; average V-O bond distance, 1.825 Å; average V-V bond distance, 3.564 Å) gradually transformed into V_2O_4 (coordination number, 6; average V-O bond distance, 1.939 Å; average V-V bond distance, 2.892 Å) and V_6O_{13} (coordination number, 6; average V-O bond distance, 1.949 Å; average V-V bond distance, 3.063 Å) with a higher V-O coordination number and averaged V-O distance as well as a lower averaged V-V distance upon NH_3 pulse reduction.

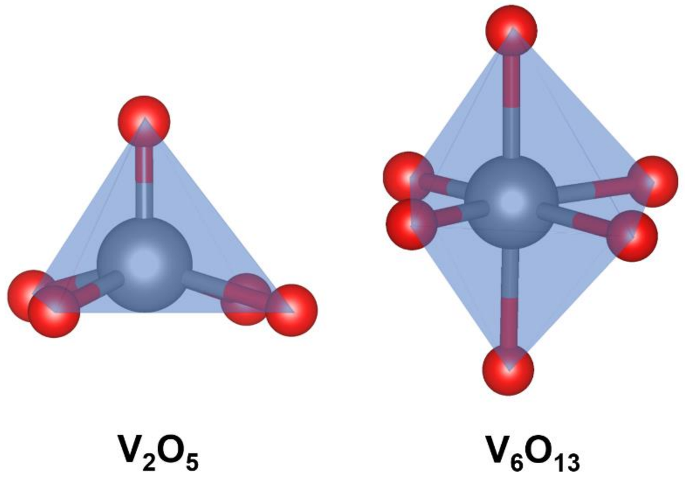

Fig. S12 The corresponding local coordination of V_2O_5 moiety, V_6O_{13} moiety, and VO_2 moiety. Silver, and red spheres depict V and O atoms, respectively.

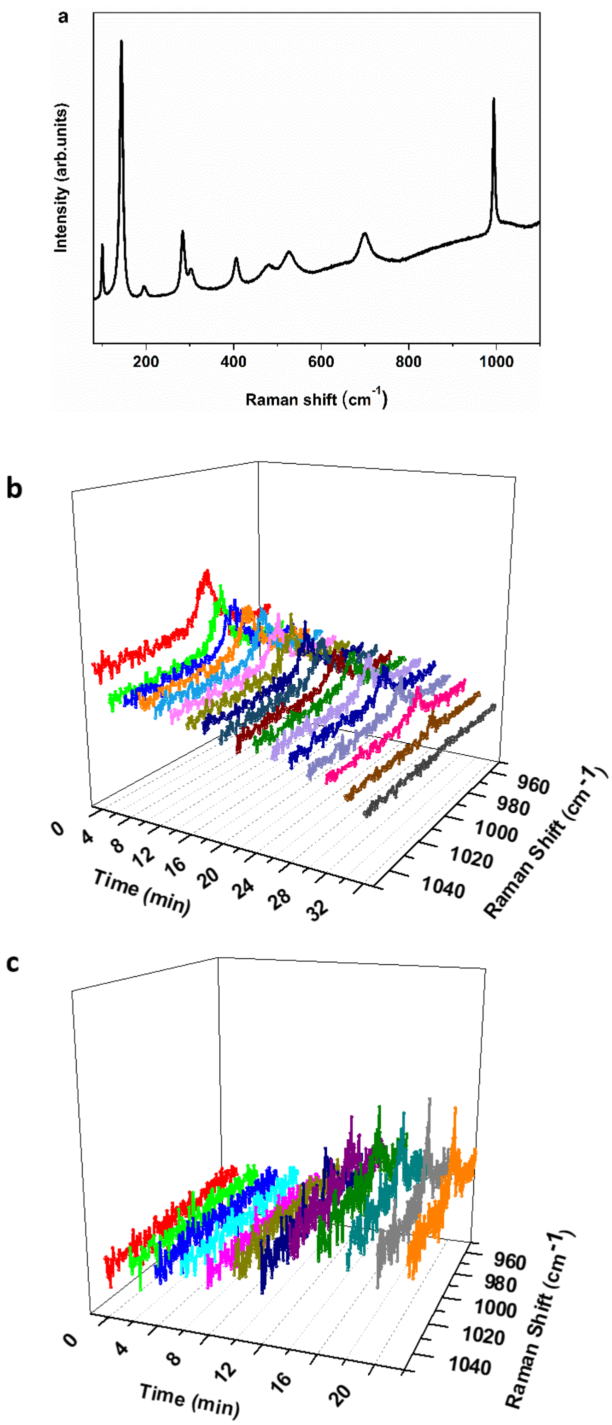

Fig. S13 (a) Raman spectrum of as prepared V_2O_5 sample collected under ambient conditions. *In situ* Raman spectra of V_2O_5 redox catalyst highlighting the evolution of V=O during the (b) reduction and (c) subsequent re-oxidation at 650 °C as a function of time. The V=O Raman peak stays at a constant shift of 994 cm^{-1} throughout the entire experiment. The vibration modes assignment are indicated. Conditions: reduction under 5% NH_3/Ar (2.2 ml/min); Oxidation under 30% O_2/Ar (2.2 ml/min).

During the reduction and oxidation process, vanadyl (V=O) vibration intensity obtained at each point was normalized respect to the vanadyl intensity at full oxidation state.

$$V{=}O \text{ relative intensity} = \frac{V{=}O\ reduced\ (oxidized)}{V{=}O\ at\ full\ oxidation\ state}$$

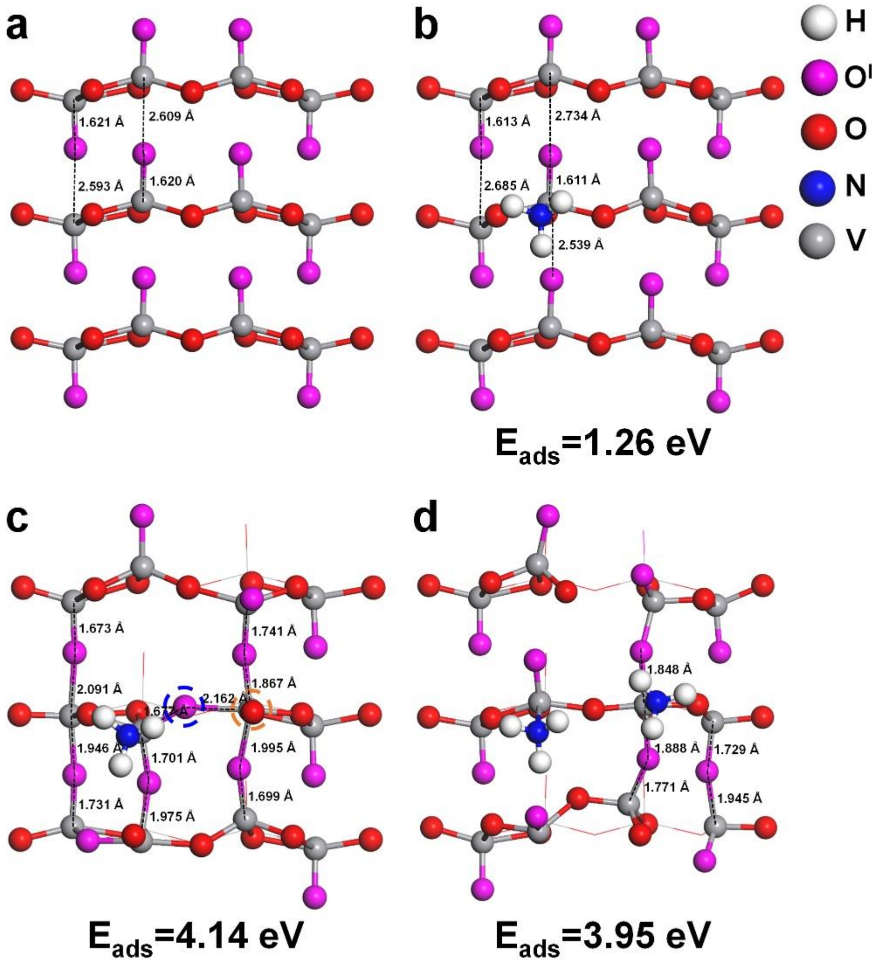

Fig. S14 Computed configurations of (a) V_2O_5 (001), (b) less stable single NH_3 adsorption, (c) more stable single NH_3, and (d) two NH_3 co-adsorption, as well as their corresponding adsorption energies. Blue dashed cycle in (c) highlights the initial O^{I}, which becomes two-coordinated after structural reconstruction, and orange dashed cycle highlights the initial O^{II}, which becomes one-coordinated after structural reconstruction.

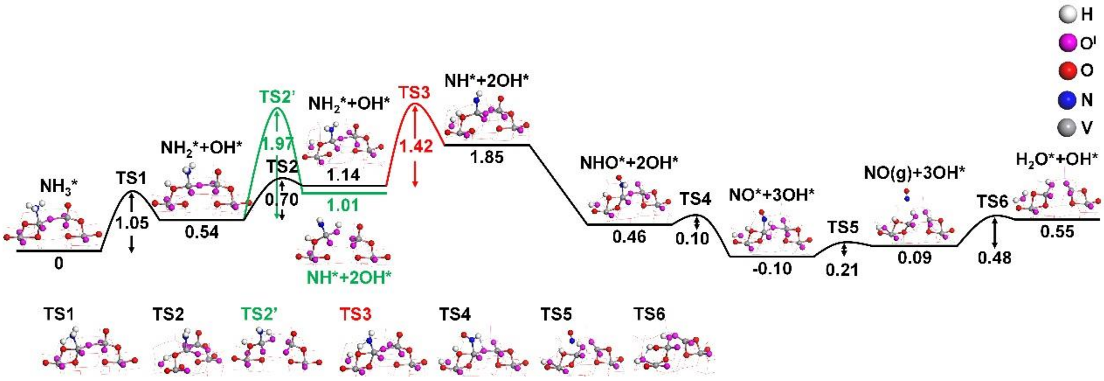

Fig. S15 Computed energy potential profiles of each elementary step for NO formation. This channel starts from the more stable single NH_3 adsorbed configuration. The kinetic barrier of each elementary step is given in eV.

It should be noted that the oxygen bonding with NH* is a two-coordinated oxygen in pristine V_2O_5 (001), however, it becomes one-coordinated after NH_3 adsorption as clarified in Fig. S14.

For a single NH_3 molecule adsorption, two stable configurations are investigated. One with a relatively weak adsorption energy (E_{ads} = 1.26 eV) that has little impact on the coordination environment of the active site (Fig. S14b). The other one has a very strong adsorption energy (E_{ads} = 4.14 eV), which causes significant surface reconstruction, inducing the mutual transformation of the adjacent one-coordinated oxygen (O^I) and two-coordinated (O^{II}) oxygen (Fig. S14c). Starting from the more stable configuration, the dehydrogenation steps is examined. As illustrated in Fig. S15, the first hydrogen transfer to adjacent two-coordinated oxygen site (O^{II}), forming an $O^{II}H$ group and NH_2* with a reaction barrier of 1.05 eV. This hydrogen tends to continue transferring to the neighboring O^I due to the much lower activation barrier (0.70 eV) as compared to direct dehydrogenation of the second hydrogen (1.97 eV). Thereby, the O^{II} site is liberated to accommodate the second hydrogen, generating NH* with a relatively high barrier of 1.42 eV, which represents the rate-determining step. Subsequently, the combination of the NH* and the O^I results in NHO* intermediate with no transition state being identified. The last hydrogen on NHO* transfers to adjacent O^{II} to form NO* with a negligible barrier of 0.10 eV, followed by the desorption step with a very small barrier of 0.21 eV. Afterwards, H_2O^I molecule can be easily formed via the hydrogen transfer from O^{II} to O^IH with a small barrier of 0.48 eV.

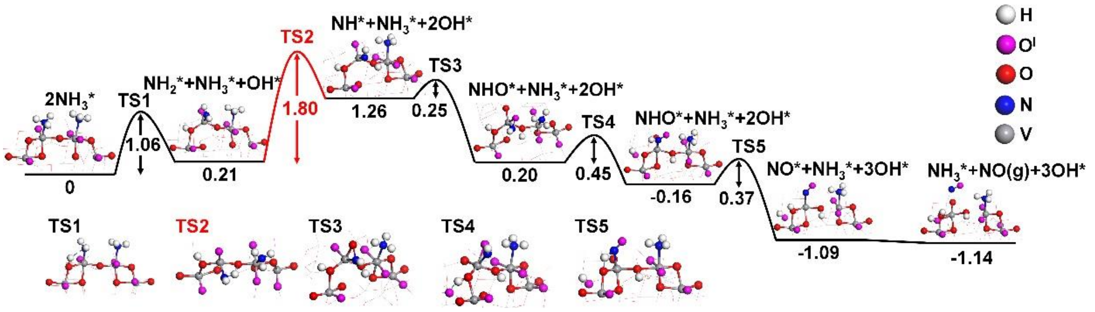

Fig. S16 Computed energy potential profiles of each elementary step for NO formation. This channel starts from the two NH_3 co-adsorbed configuration, but follows a pathway that the second dehydrogenation step occurs directly next to the first dehydrogenation step. The kinetic barrier of each elementary step is given in eV.

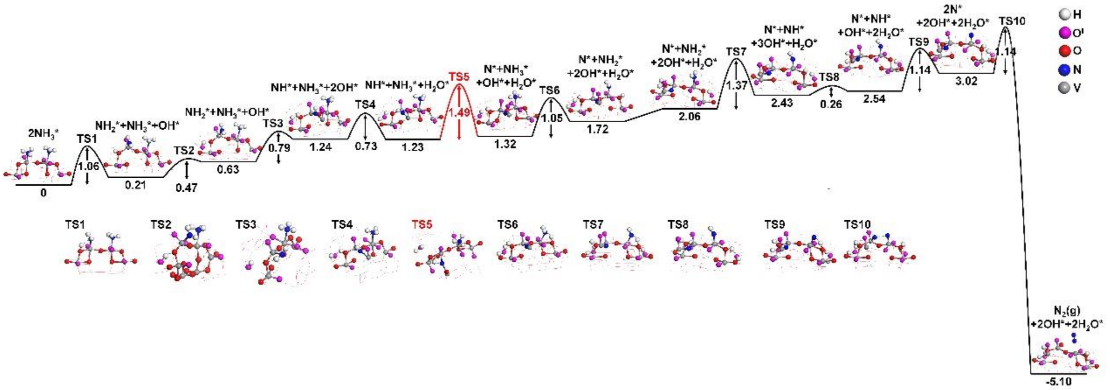

Fig. S17 Computed energy potential profiles of each elementary step for N_2 formation with the two N* as the intermediates. The kinetic barrier of each elementary step is given in eV.

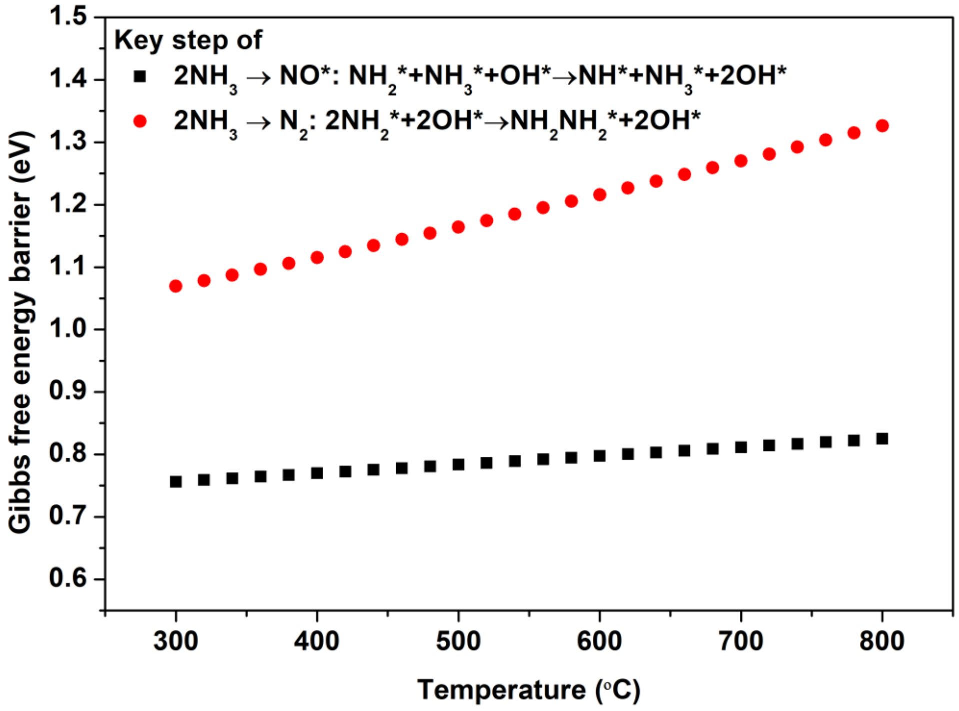

Fig. S18 Computed Gibbs free energy barriers of the key steps of NO ($NH_2^* \rightarrow NH^*$) and N_2 ($2NH_2 \rightarrow NH_2NH_2$) formations.

Table S1. Comparison of activity and selectivity for catalytic NH_3 oxidation in the proposed CLAO mode, conventional co-feed mode as well as NH_3 oxidation in the membrane mode

Redox material	Temp (^oC; red/ox)	Process	Feed gas composition	GHSV (ml g_{cat}^{-1} h^{-1}	NO production rate (ml g_{cat}^{-1} h^{-1})	NH_3 Conversion (%)	NO Selectivity (%)	N_2O Selectivity (%)	Ref.
V_2O_5	650	CLAO	5% NH_3	9,000	56.4	97.0	99.8	0.1	This work
CeO_2	900	CLAO	5% NH_3	9,000	0.9	100	12.0	0.6	This work
Fe_3O_4	750	CLAO	5% NH_3	9,000	5.64	97.3	55.8	2.7	This work
SnO_2	750	CLAO	5% NH_3	9,000	3.2	98.3	41.2	0.2	This work
MO_3	600	CLAO	5% NH_3	9,000	0.02	92.2	1.4	6.6	This work
MO_3	700	CLAO	5% NH_3	9,000	0.09	91.2	4.3	6.0	This work
WO_3	750	CLAO	5% NH_3	9,000	0	95.0	0	5.8	This work
WO_3	850	CLAO	5% NH_3	9,000	0	92.4	0	5.0	This work
WO_3	950	CLAO	5% NH_3	9,000	0	93.3	0	5.3	This work
$ZnFe_2O_4$	550	CLAO	5% NH_3	9,000	1.3	98.2	14.2	3.0	This work
$CoFe_2O_4$	650	CLAO	5% NH_3	9,000	4.8	99.5	56.3	2.0	This work
$NiFe_2O_4$	850	CLAO	5% NH_3	9,000	5.2	97.8	45.4	0.8	This work
Pt-Rh (90-95% Pt)	810-940	co-feed	12.0-12.5% NH_3	—	—	～100	92-98	1-2	ref[1]
$LaCoO_3$	700	co-feed	3% NH_3, 20% O_2	24,000	410	50	95	2	ref[2]
$LaCoO_3$	800	co-feed	3% NH_3, 20% O_2	24,000	342	60	95	1	ref[2]
$La_{0.6}Sr_{0.4}Co_{0.2}Fe_{0.8}O_{3-\delta}$	850	co-feed	1.6% NH_3, 1.6% O_2	—	—	—	77	—	ref[3]
$LaMnO_3$	870	co-feed	5% NH_3/Air	—	—	95	15.8	—	ref[4]
$LaCoO_3$	850	co-feed	5% NH_3/Air	—	—	94	10.5	—	ref[4]
$LaCuO_3$	875	co-feed	5% NH_3/Air	—	—	96	10.4	—	ref[4]
V_2O_5	650	co-feed	3.5% NH_3, 30% O_2	12,858	127.6	87.8%	22.6%	15.0%	This work
V_2O_5	650	co-feed	4.1% NH_3, 18% O_2	10,977	109.4	88.2%	16.7%	19.7%	This work
V_2O_5	650	co-feed	3.0% NH_3, 42% O_2	15,516	105.2	87.5%	15.5%	12.8%	This work
$La_{0.8}Sr_{0.2}FeO_{3-\delta}$	730-1060	Membrane	0.04-3.5% NH_3, 50% O_2	—	—	80-95	92-98	—	ref[5]
$La_{0.6}Sr_{0.4}Co_{0.2}Fe_{0.8}O_{3-\delta}$	850	Membrane	1.6% NH_3, 1.6% O_2	—	—	81	95	—	ref[3]

Table S2. Maximum oxygen removal rates from V_2O_5 redox catalyst in the first pulse reduction at various temperatures

Redox catalyst	Temperature (°C)	Maximum O^{2-} removal rate (μmol g^{-1} s^{-1})
V_2O_5	300	211.57
	400	488.47
	500	662.26
	550	676.69
	580	705.39
	600	732.27
	630	791.18
	650	862.77

Table S3. Average vanadium oxidation state determined from the overall chemical composition based on the total lattice oxygen extracted, comparison of the main edge energies (the energy measured half way up the normalized-edge step and linear combination fitting results of XANES data. The relative percentages of V^{4+} and V^{5+}, as determined from the overall chemical composition, XANES, XRD and XPS data with increasing NH_3 pulses numbers are also shown

NH_3 pulse number	Mean oxidation state[a]			Mean oxidation state[b]	Mean oxidation state[c]	XANES (bulk average)		XRD (bulk average)		XPS (surface)	
		V^{4+} (%)[a]	V^{5+} (%)[a]			V^{4+} (%)	V^{5+} (%)	V^{4+} (%)	V^{5+} (%)	V^{4+} (%)	V^{5+} (%)
pulse 30	4.89	11.16	88.84	4.97 (-)	4.93	3.22 (-)	96.78 (-)	7.00	93.00	20.28	79.72
pulse 60	4.78	21.88	78.12	4.92 (4.87)	4.91	7.83 (13.4)	92.18 (86.6)	13.90	87.10	27.67	72.33
pulse 90	4.68	32.14	67.86	4.80 (4.86)	4.81	20.13 (14.0)	79.87 (86.0)	19.00	81.00	29.46	70.54
pulse 120	4.58	42.01	57.99	4.73 (4.76)	4.66	27.26 (24.4)	72.74 (75.6)	34.00	66.00	33.54	66.46
pulse 150	4.48	51.63	48.37	4.70 (4.62)	4.60	29.38 (38.4)	70.62 (61.6)	39.60	60.40	34.32	65.68
pulse 180	4.40	60.97	39.03	4.60 (4.50)	4.56	39.57 (49.6)	60.43 (50.4)	44.40	55.60	38.38	61.62
Cycled	—	—	—	5.00	5.00	0	100	0	100	0	100

[a] The mean vanadium oxidation state is calculated from the overall chemical composition of V_2O_5 at different NH_3 pulse numbers. Meanwhile the V^{4+} and V^{5+} relatively percentage is also determined.

[b] The mean vanadium oxidation state is calculated from comparison of the main edge energies (Figure 3d) and XNAES linear combination fitting results (number shown in brackets). A slightly higher mean oxidation state is observed, which may induced by post-reaction air exposure.

[c] The mean vanadium oxidation state is calculated from the XRD Rietveld refinement. A slightly higher mean oxidation state is observed, which may induced by post-reaction air exposure.

Table S4. EXAFS fitting parameters at the V K-edge for V_2O_5 and V_2O_5 subjected to 90 pulses (V-90) and 180 pulses (V-180) of NH_3 reduction (S_0^2=0.72)

Sample	Path	R (Å)	$\sigma^2 \times 10^3$ (Å^2)	ΔE (eV)	R factor
V_2O_5	V-O	1.80±0.05	13.2±10.6	6.8±5.4	0.016
	V-V	3.35±0.06	6.6±9.4	9.8±3.1	
V-90	V-O	1.92±0.04	13.4±6.6	15.0±3.5	0.014
	V-V	3.13±0.04	2.7±6.5	8.8±2.5	
V-180	V-O	1.92±0.02	10.7±3.8	13.8±2.3	0.015
	V-V	3.12±0.05	3.4±8.5	10.0±3.1	

Supplementary References

1. L. A. I. V.A. Sadykov, I.A. Zolotarskii, L.N. Bobrova, A.S. Noskov, V.N. Parmon, E.A. Brushtein,T.V. Telyatnikova, V.I. Chernyshev, V.V. Lunin., Falter, *Appl. Catal. A*, 2000, **204**, 59-87.
2. G. Biausque and Y. Schuurman, *Journal of Catalysis*, 2010, **276**, 306-313.
3. Z. Cao, H. Jiang, H. Luo, S. Baumann, W. A. Meulenberg, H. Voss and J. Caro, *ChemCatChem*, 2014, **6**, 1190-1194.
4. L. A. Isupova, E. F. Sutormina, N. A. Kulikovskaya, L. M. Plyasova, N. A. Rudina, I. A. Ovsyannikova, I. A. Zolotarskii and V. A. Sadykov, *Catalysis Today*, 2005, **105**, 429-435.
5. J. Perez-Ramirez and B. Vigeland, *Angew. Chem., Int. Ed.*, 2005, **44**, 1112-1115.
